# State Estimation for General Complex Dynamical Networks with Incompletely Measured Information

**DOI:** 10.3390/e20010005

**Published:** 2017-12-23

**Authors:** Xinwei Wang, Guo-Ping Jiang, Xu Wu

**Affiliations:** College of Automation, Nanjing University of Posts and Telecommunications, Nanjing 210023, China

**Keywords:** state estimation, complex dynamical network, incomplete measurements

## Abstract

Estimating uncertain state variables of a general complex dynamical network with randomly incomplete measurements of transmitted output variables is investigated in this paper. The incomplete measurements, occurring randomly through the transmission of output variables, always cause the failure of the state estimation process. Different from the existing methods, we propose a novel method to handle the incomplete measurements, which can perform well to balance the excessively deviated estimators under the influence of incomplete measurements. In particular, the proposed method has no special limitation on the node dynamics compared with many existing methods. By employing the Lyapunov stability theory along with the stochastic analysis method, sufficient criteria are deduced rigorously to ensure obtaining the proper estimator gains with known model parameters. Illustrative simulation for the complex dynamical network composed of chaotic nodes are given to show the validity and efficiency of the proposed method.

## 1. Introduction

The past few decades have witnessed the rapid growth of research interests in the complex dynamical networks. Dynamical systems in real networks are abstracted into independent vertices in the complex network model, and the edges represent complicated connections between those individual systems [1]. Since the small-world [2] and scale-free [3] network models were proposed, it was possible to explore the deeper behavior in the complex dynamical networks, such as social network [4] and the Internet [5]. Additionally, based on various inner features and topological structures, the complex dynamical network has become a functional tool to describe most real networks, such as neural networks [6], transportation networks [7], electrical power grids [8], etc., [9,10]. Considerable efforts in studying complex networks have been made on the controllability and robustness analysis [11,12], synchronization and control schemes [13,14], estimation for uncertain state variables [15,16], etc. A large number of existing studies, concerning the synchronization or other problems of complex networks, have assumed that the state variables transmitted for coupling or communication could be completely measured. In fact, due to the technological limitations or massive cost for measurement, it is quite common that the state variables are partially available while the outputs are always measurable. For example, in order to control a certain circuit, acquiring the knowledge of its capacity voltages and inductance currents as many as possible is quite helpful. In reality, however, it turns out to be difficult and unwise to measure directly all the state variables. On the contrary, the outputs are always easy to be measured completely, which inspires us to make full use of output variables of the circuit to reconstruct immeasurable state variables. Therefore, estimating the uncertain state variables of complex dynamical networks with measurable outputs has become one of the hot issues for further studying.

In the traditional area of systems and control theory, several estimation techniques have been proposed over decades [17,18,19]. Recently, abundant achievements on the state estimation of complex dynamical networks have been obtained [20,21,22,23,24,25,26,27]. In order to apply to more real environments or meet certain engineering requirements, a number of unreliable or uncertain factors have been taken into account on the state estimation problem, such as coupling time delays [20,21], stochastic noisy disturbance [22], uncertain network parameters [23], incomplete measurements [24,25,26,27,28,29], etc. In this paper, we focus on the state estimation of complex dynamical networks considering incomplete measurements. In the previous studies, the Bernoulli probability distribution [24,27,28,29] was usually introduced to describe the incomplete measurements of transmitted information. Sometimes multi random independent variables were used to indicate different influencing factors in according to actual sensor saturations [25] or coupling time delays [26,27] when analyzing incomplete measurements of transmitted information. If the sent control information is incompletely measured at the receiver, the usual solution is just ignoring the incomplete measurements of information without any complements at these moments [24,25,26,27,29] or replacing them with the most recently received information [28]. The existing methods [24,25,26,27,28,29] are only effective for the complex dynamical networks whose state variables of nodes are stationary, and failure to the general complex networks whose node dynamics is generally non-stationary. The Lyapunov function in their stability analysis [24,25,26,27,28,29] was designed by error states together with node states. As a result, the error states and every node state are required to be stabilized asymptotically at the same time, which is impossible for a general complex dynamical network.

Motivated by the above discussions, we investigate the state estimation of a general complex dynamical network, and propose a novel estimator to handle the situation of incomplete measurements. When the sent output variables are received incompletely by the observer network for some time periods, the proposed estimators will replace the incompletely measured outputs with the estimated outputs during those time periods. It is a simple way to decrease the excessive deviation of the estimators caused by incomplete measurements. The novel estimation method we present has no particular restrictions on the node dynamics, even it is non-stationary. By employing the Lyapunov stability theory along with the stochastic analysis method, sufficient criteria are deduced rigorously in the form of linear matrix inequalities to obtain the proper estimator gains with known model parameters.

The rest parts of this paper are organized as follows. Problem formulation and useful preliminaries are provided in Section 2. The state estimation for a general complex dynamical network with incomplete measurements of transmitted information is further discussed in Section 3. In Section 4, illustrative simulation results are shown to verify the effectiveness of the proposed estimators. Some conclusions are drawn in Section 5.

## 2. Network Models and Preliminaries

Some necessary notations which will be used in the following are introduced here. Assume that *S*, *T* are constant matrices with proper dimensions. $∥S∥$ represents the Euclidean norm of *S*. $S^{+}$ represents the Moore-Penrose inverse of *S*. S⊗T represents the Kronecker product of *S* and *T*. *I* represents the identity matrix of proper dimensions.

Consider the general complex dynamical network composed of *N* nonidentical nodes that are fully state-coupling, which is described by

(1) $$\left\{\begin{array}{c} {\dot{x}}_{i}\left(t\right)=A_{i}x_{i}\left(t\right)+f_{i}\left(x_{i}\left(t\right)\right)+\sum_{j=1}^{N}c_{ij}\Gamma x_{j}\left(t\right),\\ y_{i}\left(t\right)=H_{i}x_{i}\left(t\right), \end{array}\right.$$

where i=1,2,…,N, $x_{i}\left(t\right)={\left[x_{i1}\left(t\right),x_{i2}\left(t\right),\cdots ,x_{in}\left(t\right)\right]}^{T}\in R^{n}$ defines the state vector of the $i^{th}$ node and $y_{i}\left(t\right)={\left[y_{i1}\left(t\right),y_{i2}\left(t\right),\cdots ,y_{im}\left(t\right)\right]}^{T}\in R^{m}$ defines the output vector of that. $A_{i}\in R^{n\times n}$ is the linear part of system matrix of the $i^{th}$ node and $f_{i}:R\times R^{n}\to R^{n}$ is a smooth nonlinear vector field of that. Both $A_{i}$ and $f_{i}$ govern the full dynamics of the $i^{th}$ node independent of interactions from the other nodes. $H_{i}\in R^{m\times n}$ is the output matrix of the $i^{th}$ node. For simplicity, it is assumed that m=1. In this way, the output $y_{i}\left(t\right)$ is a scalar as well as the linear combination of state components $\left\{x_{i1}\left(t\right),x_{i2}\left(t\right),\cdots ,x_{in}\left(t\right)\right\}$. $\Gamma \in R^{n\times n}$ is the inner coupling matrix which denotes the inner connections from node *j* to node *i*. $C={\left(c_{ij}\right)}_{N\times N}\in R^{N\times N}$ is the configuration matrix which describes the coupling strength and topological structure of the complex dynamical network. If there exists a non-zero link from node *j* to node *i* (i≠j), then $c_{ij}\neq 0$; otherwise, $c_{ij}=0$. The diagonal elements $\left\{c_{ii}\left|i=1,2,\ldots ,N\right.\right\}$ of *C* are defined to satisfy $c_{ii}=-\sum_{j=1,j\neq i}^{N}c_{ij}$.

**Figure 1:**
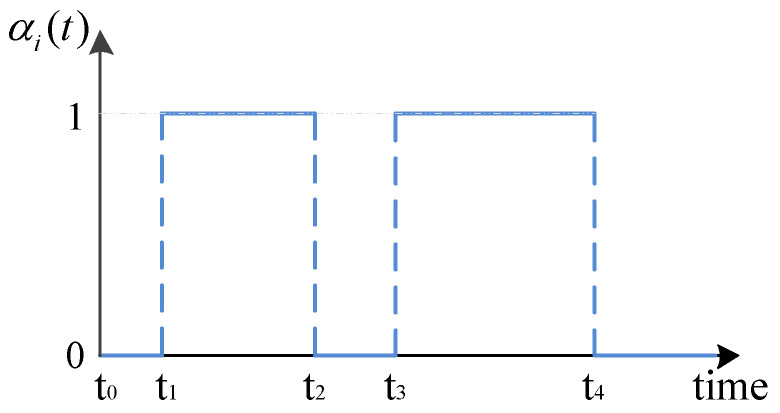
An example of continuous-time stochastic process $\alpha_{i}\left(t\right)$.

For the purpose of estimating the uncertain states which are unobservable or partly observable in the original network, one treats (1) as the original one, and establishes an observer network (2) whose evolution of node dynamics is the same as the original network [20,21,22,23,24,25,26,27].

(2) $$\left\{\begin{array}{cc} {\dot{\hat{x}}}_{i}\left(t\right) & =A_{i}{\hat{x}}_{i}\left(t\right)+f_{i}\left({\hat{x}}_{i}\left(t\right)\right)+\sum_{j=1}^{N}c_{ij}\Gamma {\hat{x}}_{j}\left(t\right)+u_{i}\left(t\right),\\ u_{i}\left(t\right) & =k_{i}\left({\hat{y}}_{i}\left(t\right)-y_{i}^{\alpha }\left(t\right)\right),\\ {\hat{y}}_{i}\left(t\right) & =H_{i}{\hat{x}}_{i}\left(t\right), \end{array}\right.$$

where ${\hat{x}}_{i}\left(t\right)={\left[{\hat{x}}_{i1}\left(t\right),{\hat{x}}_{i2}\left(t\right),\cdots ,{\hat{x}}_{in}\left(t\right)\right]}^{T}\in R^{n}$ denotes the estimated state vector of the $i^{th}$ node and ${\hat{y}}_{i}\left(t\right)\in R$ denotes the estimated output scalar of that. $u_{i}\left(t\right)$ is the state estimator imposed on the $i^{th}$ node in the observer network and $k_{i}\in R^{n}$ is the according estimator gain which needs to be determined. $y_{i}^{\alpha }\left(t\right)$ is the output variable received by the observer network (2). Compared with the original output $y_{i}\left(t\right)$ sent from the original network (1), $y_{i}^{\alpha }\left(t\right)$ is affected in some extent by the unreliable communication environments and incomplete measurements occur randomly for some time periods. In order to describe the irregularity in $y_{i}^{\alpha }\left(t\right)$, continuous-time stochastic processes $\left\{\alpha_{i}\left(t\right)\left|i=1,2,\ldots ,N\right.\right\}$ are introduced here [30].

(3) $$\left\{\begin{array}{c} \mathrm{Prob}\left\{\alpha_{i}\left(t\right)=1\right\}=E\left\{\alpha_{i}\left(t\right)\right\}={\bar{\alpha }}_{i}\left(t\right),\\ \mathrm{Prob}\left\{\alpha_{i}\left(t\right)=0\right\}=1-E\left\{\alpha_{i}\left(t\right)\right\}={\bar{\beta }}_{i}\left(t\right), \end{array}\right.$$

where $\left\{\alpha_{i}\left(t\right)\right\}$ are mutually independent and identically distributed to each other. For simplicity, ${\bar{\alpha }}_{i}\left(t\right)$, the mathematical expectation of $\alpha_{i}\left(t\right)$, is assumed to be an uncertain constant ${\bar{\alpha }}_{i}$ which is bounded by ${\bar{\alpha }}_{i}\in \left[\delta_{i1},\delta_{i2}\right]$. For example, as shown in Figure 1, $\alpha_{i}\left(t\right)=1$ represents that the output variable of the $i^{th}$ node is received completely by the observer network for the time period $t\in \left(t_{1},t_{2}\right]⋃\left(t_{3},t_{4}\right]$. Otherwise, if the output variable is received incompletely, then $\alpha_{i}\left(t\right)=0$ for the time period $t\in \left(t_{0},t_{1}\right]⋃\left(t_{2},t_{3}\right]⋃\left(t_{4},\infty \right)$. In real networks, there always exists a detecting mechanism for judging whether the sent information is received completely or not. For instance, in wireless communication networks, the attenuation coefficients of different channels could be calculate roughly by independent channel detection equipments. If the signal attenuation occurs, $\alpha_{i}\left(t\right)=0$; if not, $\alpha_{i}\left(t\right)=1$. As the channel environment changes, $\alpha_{i}\left(t\right)$ will also change and be detected at any time. Moreover, in the existing studies [24,25,26,27,28,29], $y_{i}^{\alpha }\left(t\right)$ was presented as

(4) $$y_{i}^{\alpha }\left(t\right)=\alpha_{i}\left(t\right)y_{i}\left(t\right).$$

Aiming to estimate uncertain state variables using the available output variables, it is quite helpful to obtain the complete information of the sent output variable $y_{i}\left(t\right)$ during the whole time period. Otherwise, caused by incomplete measurements, the deviation of the estimator input from the proper state is going to increase excessively. It will lead to failures of synchronization between the original and observer networks as well as the estimation of uncertain states in the original network.

To overcome the above harmful influence during the estimation process, we propose a novel estimator (5) dealing well with the incomplete measurements of transmitted output variables.

(5) $$\left\{\begin{array}{c} u_{i}\left(t\right)=k_{i}\left({\hat{y}}_{i}\left(t\right)-{\bar{y}}_{i}\left(t\right)\right),\\ {\bar{y}}_{i}\left(t\right)=\alpha_{i}\left(t\right)y_{i}\left(t\right)+\left(1-\alpha_{i}\left(t\right)\right){\hat{y}}_{i}\left(t\right). \end{array}\right.$$

For instance, as shown in Figure 1, if the output variable $y_{i}\left(t\right)$ is incompletely measured during the time period $\left(t_{2},t_{3}\right]$, the estimated output ${\hat{y}}_{i}\left(t\right)$ is used to replace $y_{i}\left(t\right)$ right for the time period $\left(t_{2},t_{3}\right]$. It will fix the excessive deviation of estimator inputs brought by the incomplete measurements in time.

Let $e_{i}\left(t\right)={\hat{x}}_{i}\left(t\right)-x_{i}\left(t\right)$, then the error dynamical network (6) could be deduced from the original network (1) and observer network (2) with the novel estimator (5).

(6) $$\begin{array}{cc} {\dot{e}}_{i}\left(t\right) & ={\dot{\hat{x}}}_{i}\left(t\right)-{\dot{x}}_{i}\left(t\right)\\ & =A_{i}e_{i}\left(t\right)+f_{i}\left({\hat{x}}_{i}\left(t\right)\right)-f_{i}\left(x_{i}\left(t\right)\right)+\sum_{j=1}^{N}c_{ij}\Gamma e_{j}\left(t\right)+\alpha_{i}\left(t\right)k_{i}H_{i}e_{i}\left(t\right). \end{array}$$

In order to stabilize the error dynamical network (6) and reconstruct the uncertain state variables in the original network (1), one suitable assumption and two useful lemmas are introduced as follows. We assume that functions $\left\{f_{i}\left(\cdot \right)\left|i=1,2,\ldots ,N\right.\right\}$ are continuous and satisfy the following condition: there exist positive constants $\left\{\mu_{i}\left|i=1,2,\ldots ,N\right.\right\}$ such that

(7) $$∥f_{i}\left(z_{1}\left(t\right)\right)-f_{i}\left(z_{2}\left(t\right)\right)∥\leq \mu_{i}∥z_{1}\left(t\right)-z_{2}\left(t\right)∥,$$

which hold for any vectors $z_{1}\left(t\right)$, $z_{2}\left(t\right)\in R^{n}$.

**Lemma** **1.** For any vectors $z_{1}\left(t\right)$, $z_{2}\left(t\right)\in R^{n}$, the inequality $2z_{1}^{T}\left(t\right)z_{2}\left(t\right)\leq z_{1}^{T}\left(t\right)z_{1}\left(t\right)+z_{2}^{T}\left(t\right)z_{2}\left(t\right)$ holds for any t.

**Lemma** **2** ([31])**.** *Suppose that there is a matrix $S=\left[\begin{array}{cc} S_{11} & S_{12}\\ S_{21} & S_{22} \end{array}\right]$ satisfying $S_{11}=S_{11}^{T}$, $S_{22}=S_{22}^{T}$ and $S_{12}=S_{21}^{T}$. The condition S<0 is equivalent to $S_{22}<0$ and $S_{11}-S_{12}S_{22}^{-1}S_{21}<0$.*

## 3. Main Results

In this section, based on the stabilization of the error dynamical network (6) from the original and observer networks, the main results of estimation of uncertain state variables with random incomplete measurements of transmitted output variables are shown as follows.

**Theorem** **1.** *Suppose that the assumption (7) holds. If the considered matrix* Ψ *satisfies the following inequality*

(8) $$\Psi =\left[\begin{array}{cc} \Pi & P\\ P & -I \end{array}\right]<0,$$

*where*

$$\begin{array}{cc} \Pi & =PA+A^{T}P+\mu^{2}I+\alpha M+\alpha M^{T}+P\left(C⊗\Gamma \right)+\left(C^{T}⊗\Gamma^{T}\right)P, \end{array}$$

*then the error dynamical network (6) will be stabilized to the origin, so that the original network (1) and observer network (2) will synchronize asymptotically. The uncertain state variables $x_{i}\left(t\right)$ in the original network (1) will be reconstructed by ${\hat{x}}_{i}\left(t\right)$ eventually, i.e.*

$$\lim_{t\to \infty }\;∥e_{i}\left(t\right)∥=\lim_{t\to \infty }\;∥{\hat{x}}_{i}\left(t\right)-x_{i}\left(t\right)∥=0,$$

*where $P_{i}=P_{i}^{T}>0$, $P=diag\left(P_{1},P_{2},\ldots ,P_{N}\right)$, $A=diag\left(A_{1},A_{2},\ldots ,A_{N}\right)$, $\mu =diag\left(\mu_{1},\mu_{2},\ldots ,\mu_{N}\right)⊗I$, $\alpha =diag\left(\delta_{11},\delta_{21},\ldots ,\delta_{N1}\right)⊗I$, $M=diag\left(M_{1},M_{2},\ldots ,M_{N}\right)=diag\left(P_{1}k_{1}H_{1},P_{2}k_{2}H_{2},\ldots ,P_{N}k_{N}H_{N}\right)$, the $i^{th}$ estimator gain $k_{i}$ is obtained by $k_{i}=P_{i}^{-1}M_{i}H_{i}^{+}$.*

**Proof of** **Theorem 1.** Choose the scalar Lyapunov function *V* as follows.

(9) $$V=\sum_{i=1}^{N}e_{i}^{T}\left(t\right)P_{i}e_{i}\left(t\right).$$

The derivative of *V* taking the form of mathematical expectation is calculated in (10) along with the estimator (5), and one has

(10) $$\begin{array}{cc} E(\dot{V})= & \sum_{i=1}^{N}\left(e_{i}^{T}\left(t\right)P_{i}{\dot{e}}_{i}\left(t\right)+{\dot{e}}_{i}^{T}\left(t\right)P_{i}e_{i}\left(t\right)\right)\\ = & \sum_{i=1}^{N}e_{i}^{T}\left(t\right)P_{i}\left(A_{i}e_{i}\left(t\right)+f_{i}\left({\hat{x}}_{i}\left(t\right)\right)-f_{i}\left(x_{i}\left(t\right)\right)\right)+\sum_{i=1}^{N}e_{i}^{T}\left(t\right)P_{i}\left(\sum_{j=1}^{N}c_{ij}\Gamma e_{j}\left(t\right)+{\bar{\alpha }}_{i}k_{i}H_{i}e_{i}\left(t\right)\right)\\ & +\sum_{i=1}^{N}{\left(A_{i}e_{i}\left(t\right)+f_{i}\left({\hat{x}}_{i}\left(t\right)\right)-f_{i}\left(x_{i}\left(t\right)\right)\right)}^{T}P_{i}e_{i}\left(t\right)+\sum_{i=1}^{N}{\left(\sum_{j=1}^{N}c_{ij}\Gamma e_{j}\left(t\right)+{\bar{\alpha }}_{i}k_{i}H_{i}e_{i}\left(t\right)\right)}^{T}P_{i}e_{i}\left(t\right), \end{array}$$

Together with the assumption (7) and Lemma 1, one gets

(11) $$\begin{array}{cc} E(\dot{V})= & \sum_{i=1}^{N}e_{i}^{T}\left(t\right)\left(P_{i}A_{i}+A_{i}^{T}P_{i}\right)e_{i}\left(t\right)+\sum_{i=1}^{N}e_{i}^{T}\left(t\right)\left({\bar{\alpha }}_{i}P_{i}k_{i}H_{i}+{\bar{\alpha }}_{i}H_{i}^{T}k_{i}^{T}P_{i}\right)e_{i}\left(t\right)\\ & +\sum_{i=1}^{N}e_{i}^{T}\left(t\right)P_{i}\left(f_{i}\left({\hat{x}}_{i}\left(t\right)\right)-f_{i}\left(x_{i}\left(t\right)\right)\right)+\sum_{i=1}^{N}{\left(f_{i}\left({\hat{x}}_{i}\left(t\right)\right)-f_{i}\left(x_{i}\left(t\right)\right)\right)}^{T}P_{i}e_{i}\left(t\right)\\ & +\sum_{i=1}^{N}\sum_{j=1}^{N}e_{i}^{T}\left(t\right)P_{i}c_{ij}\Gamma e_{j}\left(t\right)+{\sum_{i=1}^{N}\left(\sum_{j=1}^{N}c_{ij}\Gamma e_{j}\left(t\right)\right)}^{T}P_{i}e_{i}\left(t\right)\\ \leq & \sum_{i=1}^{N}e_{i}^{T}\left(t\right)\left(P_{i}A_{i}+A_{i}^{T}P_{i}\right)e_{i}\left(t\right)+\sum_{i=1}^{N}e_{i}^{T}\left(t\right)\left({\bar{\alpha }}_{i}P_{i}k_{i}H_{i}+{\bar{\alpha }}_{i}H_{i}^{T}k_{i}^{T}P_{i}\right)e_{i}\left(t\right)\\ & +\sum_{i=1}^{N}e_{i}^{T}\left(t\right)\left(P_{i}P_{i}^{T}+\mu_{i}^{2}I\right)e_{i}\left(t\right)+\sum_{i=1}^{N}\sum_{j=1}^{N}e_{i}^{T}\left(t\right)P_{i}c_{ij}\Gamma e_{j}\left(t\right)+{\sum_{i=1}^{N}\left(\sum_{j=1}^{N}c_{ij}\Gamma e_{j}\left(t\right)\right)}^{T}P_{i}e_{i}\left(t\right)\\ = & \sum_{i=1}^{N}e_{i}^{T}\left(t\right)\left(P_{i}A_{i}+A_{i}^{T}P_{i}+P_{i}P_{i}^{T}+\mu_{i}^{2}I+{\bar{\alpha }}_{i}P_{i}k_{i}H_{i}+{\bar{\alpha }}_{i}H_{i}^{T}k_{i}^{T}P_{i}\right)e_{i}\left(t\right)\\ & +\sum_{i=1}^{N}\sum_{j=1}^{N}e_{i}^{T}\left(t\right)P_{i}c_{ij}\Gamma e_{j}\left(t\right)+{\sum_{i=1}^{N}\left(\sum_{j=1}^{N}c_{ij}\Gamma e_{j}\left(t\right)\right)}^{T}P_{i}e_{i}\left(t\right). \end{array}$$

Let $e\left(t\right)={\left[e_{1}^{T}\left(t\right),e_{2}^{T}\left(t\right),\ldots ,e_{N}^{T}\left(t\right)\right]}^{T}\in R^{N}$, and one obtains

(12) $$E\left(\dot{V}\right)\leq e^{T}\left(t\right)\Omega e\left(t\right)$$

where

$$\begin{array}{cc} \Omega & =PA+A^{T}P+PP+\mu^{2}I+\alpha M+\alpha M^{T}+P\left(C⊗\Gamma \right)+\left(C^{T}⊗\Gamma^{T}\right)P. \end{array}$$

According to Lemma 2, one could further transform the matrix Ω into the equivalent LMI condition (8). Taking Ψ<0 from (8), one has $E(\dot{V})<0$ holding for any e(t)≠0. Only if e(t)=0, then $E(\dot{V})=0$. Based on the Lyapunov stability theory, the error dynamical network (6) is asymptotically stable at the origin, that means the uncertain state variables in the original network (1) are reconstructed successfully by the novel estimator (5) dealing with the incomplete measurements of the output information. The proof is completed. ☐

## 4. Numerical Simulations

**Figure 2:**
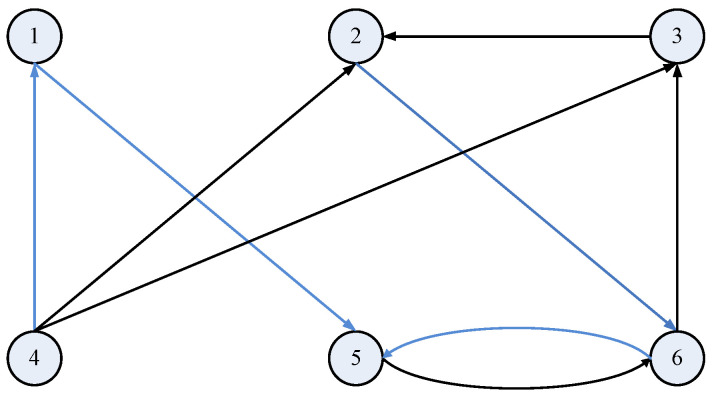
Topological structure of the original network (14).

In this section, some numerical examples are presented to illustrate the effectiveness of the novel estimator that we proposed. The chaotic Lorenz system is selected to characterize the node dynamics shown as (13). The Lorenz system is one of the most well-known chaotic systems, of which the irregular behavior could increase the difficulty of synchronization, so that it could further verify the effectiveness of the proposed method.

(13) $$\left\{\begin{array}{c} {\dot{x}}_{i1}\left(t\right)=a\left(x_{i2}\left(t\right)-x_{i1}\left(t\right)\right),\\ {\dot{x}}_{i2}\left(t\right)=cx_{i1}\left(t\right)-x_{i2}\left(t\right)-x_{i1}\left(t\right)x_{i3}\left(t\right),\\ {\dot{x}}_{i3}\left(t\right)=x_{i1}\left(t\right)x_{i2}\left(t\right)-bx_{i3}\left(t\right). \end{array}\right.$$

when a=10, b=8/3, c=28, the Lorenz system shows the chaotic behavior. Due to the bounded chaotic attractors in a certain region [32,33], the assumption (7) is evidently satisfied in the Lorenz system. For brevity, we consider a complex dynamical network consisting of six identical nodes in order to validate the above theoretical results. We assume that the inner coupling matrix Γ=I, and all $\left\{H_{i}\left|i=1,2,\ldots ,6\right.\right\}$ are assumed to be the same as the matrix *H*, which is shown as follows.

(14) $$\left\{\begin{array}{c} {\dot{x}}_{i}\left(t\right)=Ax_{i}\left(t\right)+f\left(x_{i}\left(t\right)\right)+\sum_{j=1}^{6}c_{ij}\Gamma x_{j}\left(t\right),\\ y_{i}\left(t\right)=Hx_{i}\left(t\right), \end{array}\right.$$

where $A=\left[\begin{array}{ccc} -10 & 10 & 0\\ 28 & -1 & 0\\ 0 & 0 & -8/3 \end{array}\right]$, $\Gamma =\left[\begin{array}{ccc} 1 & 0 & 0\\ 0 & 1 & 0\\ 0 & 0 & 1 \end{array}\right]$, $H=\left[\begin{array}{ccc} 2 & 1 & 1 \end{array}\right]$. The topological structure of the network (14) is denoted by the matrix *C* which is shown as Figure 2.

$$C={\left(c_{ij}\right)}_{6\times 6}=\left[\begin{array}{cccccc} -2 & 0 & 0 & 2 & 0 & 0\\ 0 & -2 & 1 & 1 & 0 & 0\\ 0 & 0 & -2 & 1 & 0 & 1\\ 0 & 0 & 0 & 0 & 0 & 0\\ 2 & 0 & 0 & 0 & -4 & 2\\ 0 & 2 & 0 & 0 & 1 & -3 \end{array}\right].$$

Receiving the node dynamics of the considered network (14) which is treated as the original one, the observer network (15) is established with the incompletely measured output information $y_{i}^{\alpha }\left(t\right)$.

(15) $$\left\{\begin{array}{cc} {\dot{\hat{x}}}_{i}\left(t\right) & =A_{i}{\hat{x}}_{i}\left(t\right)+f_{i}\left({\hat{x}}_{i}\left(t\right)\right)+\sum_{j=1}^{6}c_{ij}\Gamma {\hat{x}}_{j}\left(t\right)+u_{i}\left(t\right),\\ u_{i}\left(t\right) & =k_{i}\left({\hat{y}}_{i}\left(t\right)-{\bar{y}}_{i}\left(t\right)\right),\\ {\hat{y}}_{i}\left(t\right) & =H{\hat{x}}_{i}\left(t\right),\\ y_{i}^{\alpha }\left(t\right) & =\alpha_{i}\left(t\right)y_{i}\left(t\right),\\ {\bar{y}}_{i}\left(t\right) & =y_{i}^{\alpha }\left(t\right)+\left(1-\alpha_{i}\left(t\right)\right){\hat{y}}_{i}\left(t\right), \end{array}\right.$$

The mathematical expectations of stochastic processes $\left\{\alpha_{i}\left(t\right)\left|i=1,2,\ldots ,6\right.\right\}$ are assumed to be the same, uncertain but bounded by ${\bar{\alpha }}_{i}=\bar{\alpha }\in \left(\delta_{1},\delta_{2}\right)=\left(0.6,0.8\right)$ for brevity. Resorting to the LMI toolbox of MATLAB, one could get a feasible solution by solving the matrix inequality (8), which is shown in the following.

$$\begin{array}{c} P_{1}=\left[\begin{array}{ccc} 26.4980 & -1.6922 & 0\\ -1.6922 & 19.7662 & 0\\ 0 & 0 & 25.7098 \end{array}\right],P_{2}=\left[\begin{array}{ccc} 26.4775 & -1.6877 & 0\\ -1.6877 & 19.8545 & 0\\ 0 & 0 & 25.7999 \end{array}\right],\\ P_{3}=\left[\begin{array}{ccc} 26.4616 & -1.6644 & 0\\ -1.6644 & 19.8910 & 0\\ 0 & 0 & 25.7516 \end{array}\right],P_{4}=\left[\begin{array}{ccc} 27.2440 & -2.1692 & 0\\ -2.1692 & 19.2774 & 0\\ 0 & 0 & 26.7690 \end{array}\right],\\ P_{5}=\left[\begin{array}{ccc} 26.2775 & -1.3786 & 0\\ -1.3786 & 20.5666 & 0\\ 0 & 0 & 27.2213 \end{array}\right],P_{6}=\left[\begin{array}{ccc} 26.3732 & -1.5536 & 0\\ -1.5536 & 20.1013 & 0\\ 0 & 0 & 26.8136 \end{array}\right],\\ k_{1}=\left[\begin{array}{c} -49.4383\\ -69.7363\\ -43.3924 \end{array}\right],k_{2}=\left[\begin{array}{c} -49.4285\\ -69.4809\\ -43.2633 \end{array}\right],k_{3}=\left[\begin{array}{c} -49.3838\\ -69.3209\\ -43.2961 \end{array}\right],\\ k_{4}=\left[\begin{array}{c} -50.3987\\ -73.4525\\ -41.9269 \end{array}\right],k_{5}=\left[\begin{array}{c} -48.2936\\ -65.6912\\ -40.1592 \end{array}\right],k_{6}=\left[\begin{array}{c} -48.9326\\ -67.8944\\ -41.3151 \end{array}\right]. \end{array}$$

The initial values of state variables in the original and observer networks are chosen randomly in the interval (0,1).

**Figure 3:**
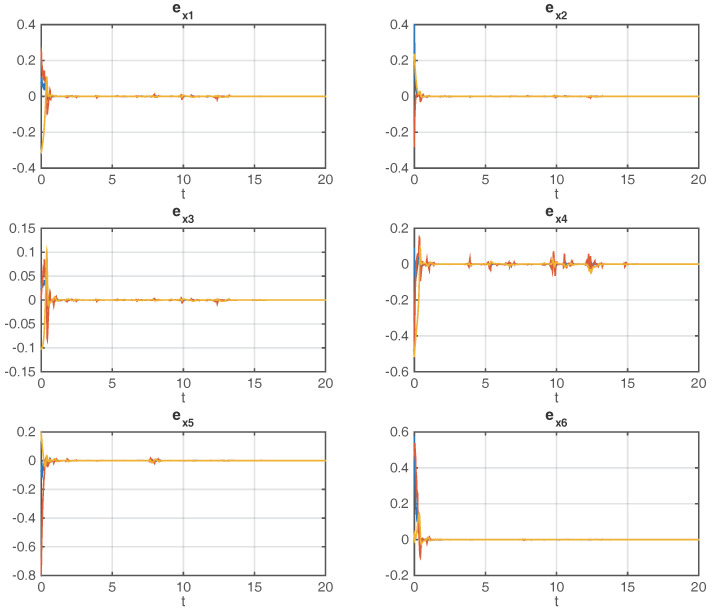
Dynamical error variables between corresponding nodes $\left\{x_{i}\left|i=1,2,\ldots ,6\right.\right\}$ in the original and observer networks.

Figure 3 shows the reconstructing process of uncertain state variables in the considered network (14), which is conducted by the observer network (15).

**Figure 4:**
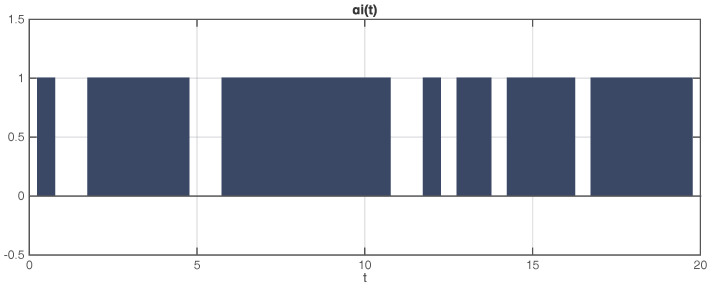
Diagram of the stochastic process $\alpha_{i}\left(t\right)$ versus time *t*.

From Figure 3, it is obvious to tell that the dynamical error variables between corresponding nodes in the original and observer networks just take a little time to converge to zero under the influence of random incomplete measurements of transmitted output variables. The evolution of the stochastic process $\alpha_{i}\left(t\right)$ is shown in Figure 4, which indicates a common situation of incomplete measurements in the unreliable communication channel.

Figure 3 and Figure 4 illustrate that, just using the scalar output variables $\left\{y_{i}\left(t\right)\right\}$, the uncertain state vectors $\left\{x_{i}\left(t\right)\right\}$ in the considered network are rapidly followed by the corresponding state vectors $\left\{{\hat{x}}_{i}\left(t\right)\right\}$ in the observer network, in spite of the incomplete measurements characterized by random variable $\alpha_{i}\left(t\right)$, which demonstrates that the designed estimators perform well dealing with incomplete measurements.

**Remark** **1.** The existing methods [24,25,26,27,28,29] require that the node dynamics must reach the stationary state by itself. If this condition is not met, the results with existing methods are the failure of state estimation, which means trajectories of estimated state variables would not follow ones in the original network.

## 5. Conclusions

Reconstructing uncertain state variables of general complex dynamical networks with randomly incomplete measurements of transmitted information has been studied in this paper. The random incomplete measurements can prevent the successful state estimation process. Different from previous researches, our novel method is able to balance the excessively deviated estimators and performs well under the influence of incomplete measurements. Especially, there is no special limitation on the node dynamics. By employing the Lyapunov stability theory with the stochastic analysis method, mathematical derivation of the mechanism are deduced rigorously to obtain sufficient criteria in terms of an LMI approach with known model parameters. Illustrative examples are given to show the effectiveness of our proposed method.
